# NS1 Specific CD8^+^ T-Cells with Effector Function and TRBV11 Dominance in a Patient with Parvovirus B19 Associated Inflammatory Cardiomyopathy

**DOI:** 10.1371/journal.pone.0002361

**Published:** 2008-06-04

**Authors:** Mathias Streitz, Michel Noutsias, Rudolf Volkmer, Maria Rohde, Gordon Brestrich, Andrea Block, Katrin Klippert, Katja Kotsch, Bernhard Ay, Michael Hummel, Uwe Kühl, Dirk Lassner, Heinz-Peter Schultheiss, Hans-Dieter Volk, Florian Kern

**Affiliations:** 1 Institute of Medical Immunology, Charité–Universitätsmedizin Berlin, Berlin, Germany; 2 Department of Cardiology and Pneumonology, Charité–Universitätsmedizin Berlin, Berlin, Germany; 3 Institute of Pathology, Charité–Universitätsmedizin Berlin, Berlin, Germany; 4 Institute of Cardiac Diagnostic and Therapy, Berlin, Germany; 5 Division of Medicine, Brighton and Sussex Medical School, Brighton, United Kingdom; University of California San Francisco, United States of America

## Abstract

**Background:**

Parvovirus B19 (B19V) is the most commonly detected virus in endomyocardial biopsies (EMBs) from patients with inflammatory cardiomyopathy (DCMi). Despite the importance of T-cells in antiviral defense, little is known about the role of B19V specific T-cells in this entity.

**Methodology and Principal Findings:**

An exceptionally high B19V viral load in EMBs (115,091 viral copies/μg nucleic acids), peripheral blood mononuclear cells (PBMCs) and serum was measured in a DCMi patient at initial presentation, suggesting B19V viremia. The B19V viral load in EMBs had decreased substantially 6 and 12 months afterwards, and was not traceable in PBMCs and the serum at these times. Using pools of overlapping peptides spanning the whole B19V proteome, strong CD8^+^ T-cell responses were elicited to the 10-amico-acid peptides SALKLAIYKA (19.7% of all CD8^+^ cells) and QSALKLAIYK (10%) and additional weaker responses to GLCPHCINVG (0.71%) and LLHTDFEQVM (0.06%). Real-time RT-PCR of IFNγ secretion-assay-enriched T-cells responding to the peptides, SALKLAIYKA and GLCPHCINVG, revealed a disproportionately high T-cell receptor Vbeta (TRBV) 11 expression in this population. Furthermore, dominant expression of type-1 (IFNγ, IL2, IL27 and T-bet) and of cytotoxic T-cell markers (Perforin and Granzyme B) was found, whereas gene expression indicating type-2 (IL4, GATA3) and regulatory T-cells (FoxP3) was low.

**Conclusions:**

Our results indicate that B19V Ag-specific CD8^+^ T-cells with effector function are involved in B19V associated DCMi. In particular, a dominant role of TRBV11 and type-1/CTL effector cells in the T-cell mediated antiviral immune response is suggested. The persistence of B19V in the endomyocardium is a likely antigen source for the maintenance of CD8^+^ T-cell responses to the identified epitopes.

## Introduction

Ever since its first description [1], Parvovirus B19 (B19V) has been associated with various human diseases, including erythema infectiosum, hydrops fetalis, anemia, aplastic crisis and arthritis, vasculitis [2], [3], [4]. B19V contains a single-stranded DNA genome which replicates in the nucleus of infected host cells. The icosahedral capsid of B19V consists of two structural proteins, VP1 (83 kDa) and VP2 (58 kDa), which are encoded by the same reading frame. VP1 differs from VP2 only by an additional N-terminal fragment. The nonstructural protein 1 (NS1) is required for virus replication and propagation in the host cell [2].

B19V is the most commonly detected virus in endomyocardial biopsies (EMBs) from patients presenting with acute myocarditis (AMC), dilated cardiomyopathy (DCM) or EMBs proven inflammatory cardiomyopathy (DCMi) [5], [6], [7], [8].

Despite the importance of T-cells in antiviral defense, little is known about anti-B19V specific T-cells in this entity. Using peptide pools and intracellular IFNγ secretion as read-out, antigen specific T-cells have been identified in other diseases [9], [10]. Here, we provide evidence for antigen-specific T-cells in a patient with EMBs proven DCMi and exceptionally high B19V viral load at the initial presentation. We furthermore characterized T-cell receptor Vbeta (TRBV) expression and the expression profile of type 1, type 2, cytotoxic and regulatory T-cell markers.

## Methods

### Patient description

A previously healthy 44 year-old man was admitted to our department with progressive heart failure after a prior respiratory tract infection with febrile temperatures up to 39°C three weeks ago without elevated biochemical markers for myocardial ischemia. Coronary artery disease was excluded by coronary angiography. Left ventricular ejection fraction (LVEF) assessed by echocardiography according to the modified Simpson's method was 45%. This clinical presentation was consistent with the tentative clinical diagnosis of acute myocarditis [6], [11]. Cardiac MRI for non-invasive detection of myocardial edema was not possible in this patient because he had a hip implant owing to a traffic accident 8 years ago. There was no evidence for rheumatic diseases in his past medical history. The patient was treated with heart failure medication (carvedilol, ramipril, torasemide and spironolactone). LVEF gradually increased, and LV end-diastolic diameter (LVEDD) gradually decreased at echocardiographic follow-up analyses (Table 1).

**Table 1:** Course of clinical parameters, EMBs and serological results.

	Initial presentation	6 months	12 months
**LVEF (%)**	45	54	70
**LVEDD (mm)**	64	56	54
**EMBs - B19V nPCR/EMBs - B19V qPCR**	positive	positive	positive
	115,091	23,846	1,026
**PBMCs - B19V nPCR/PBMCs - B19V qPCR**	positive	negative	negative
	184,000		
**Serum - B19V nPCR/Serum–B19V qPCR**	positive	negative	negative
	57,468		
**B19V IgG titer (ELISA)**	146	143.6	141.7
**B19V IgG fractions (** ***recom*** **Line® blot)**	VP-2p; VP-N; VP-1S; VP-2r; VP-C	VP-2p; VP-N; VP-1S; VP-2r; VP-C; NS1	VP-2p; VP-N; VP-1S; VP-2r; VP-C; NS1
**B19V IgM titer (ELISA)**	115.7	negative	negative
**B19V IgM fractions (** ***recom*** **Line® blot)**	VP-2p; VP-N; VP-1S; VP-2r; VP-C	negative	negative
**CD3/mm^2^**	16	7.5	3.6
**LFA-1/mm^2^**	36.4	12.2	7.4
**CD45R0/mm^2^**	19	10.1	5.5
**Mac-1/mm^2^**	36.8	14.8	13.3
**HLA class I AF (%)**	10.4	7.4	5.1
**ICAM-1 AF (%)**	4.1	2.7	1.1

Evolution of echocardiographic parameters (LVEF, LVEDD: LV enddiastolic diameter), of the B19V quantification results in EMBs and PBMCs (viral copies/μg nucleic acids in EMBs, and viral copies/ml serum, respectively) and of the DIA quantified, immunohistologically marked infiltrates and CAMs expression in EMBs. AF: fraction of area in percent (DIA derived value for CAMs expression). Normal values for DIA quantified infiltrates and CAMs expression in EMBs: CD3: <7/mm^2^, LFA-1: <9/mm^2^, CD45R0: <7/mm^2^, Mac-1: <35/mm^2^, HLA class I: <5.5%, ICAM-1: <1.2%.

### Description of Investigations undertaken

Detection and quantification of B19V in EMBs, PBMCs and serum; quantification of intramyocardial inflammation in EMBs; detection of anti-B19V-IgG, anti-B19V-IgM and characterization of anti-B19V humoral response

6 EMBs (1 for histological, 1 for immunohistological, and 4 for nPCR analyses of viral genomes) were obtained from the right ventricular septum at each time point by a standard right femoral venous approach. Five ml of blood was obtained in Vacutainer® EDTA tubes (BD Biosciences, Heidelberg, Germany). EMBs, PBMCs and serum were examined simultaneously for viral genomes. Nested PCR (nPCR) for viral genomes was carried out as described in detail elsewhere [6]. In brief, 2 EMBs were subjected to RNA extraction by Trizol® (Invitrogen, Karlsruhe, Germany) for nested PCR (nPCR) of enteroviruses, and 2 EMBs were subjected to DNA extraction (Puregene mousetail DNA extraction kit, Qiagen, Hilden, Germany) for B19V, human herpes virus type 6, adenovirus and Epstein-Barr virus. Each time 2 EMBs were pooled to minimize sampling error effects as a routine procedure in our laboratory. Single EMBs were not analyzed. For B19V nPCR and qPCR the methods are described in brief as follows: DNA extracted from EMBs, PBMCs and serum, respectively, was amplified using B19V-specific primers yielding a product of 290 bp in the first and of 173 bp in the second round. Direct sequencing was carried out with an 8-capillary sequencing system CEQ 8000 (Beckman-Coulter, Krefeld, Germany). The sequences were aligned with the archived B19V genome (NCBI GenBank accession No. AY386330) using the Phylogenetic Data Editor (PHYDE) Software (Institute for Botanics, Dresden/Bonn, Germany). The primers and probe for B19V quantitative PCR (qPCR) were designed from the B19V VP1/2 open reading frame (sequences given in Table 2). After initial denaturation at 95°C for 10 min, amplification was carried out by 38 two-step cycles (15 sec at 95°C, 1 min at 57°C) on a 7900 HT Fast Real-Time PCR System (Applied Biosystems/ABI, Darmstadt, Germany). Serial dilutions (3.5 to 3.5×10^4^ genomes per assay) of B19V plasmid DNA (Genexpress, Berlin, Germany) were simultaneously coamplified to generate a standard curve. Subsequent calculation of viral loads was performed based on the ratio of the estimated viral genome copy numbers in the TaqMan assay over the amount of incorporated human genomic DNA for EMBs and PBMCs, respectively, while the calculation of viral loads in the serum was normalized to milliliters of serum volume. Concentrations of isolated genomic human DNA amounts were quantitatively measured using the Quantifiler Human DNA quantification kit (ABI) according to manufacturer's instructions. Quantification of immunohistologically stained cells and cell adhesion molecules (CAMs) in cryosections of EMBs was carried out as described in detail elsewhere [12], [13].

**Table 2:** Primers and probes of self-designed gene expression assays.

Gene name	5′ sense 3′ primer	5′ antisense 3′ primer	Fluorescence hybridization probe
TRBC	TCCGCTGTCAAGTCCAGTTCTA	GACAGGACCCCTTGCTGGTA	ACGAGTGGACCCAGGATAGGGCCAA
TRBV common antisense primer		CTGCTTCTGATGGCTCAAACA	
TRBV common probe			CACCCGAGGTCGCT
TRBV2	ACTCTGAAGATCCGGTCCACAA		
TRBV3	ATCAATTCCCTGGAGCTTGGT		
TRBV4	CCTGAATGCCCCAACAGC		
TRBV5	GCTCTGAGCTGAATGTGAACGC		
TRBV5WBL	GCTCTGAGATGAATGTGAGTGC		
TRBV6	CACTGACAAAGGAGAAGTCCC		
TRBV6WBL6-2	AACTGCCAAAGGAGAGGTCCC		
TRBV6WBL6-4	CACTGGCAAAGGAGAAGTCCC		
TRBV7	CTCTCAGGTGTGATCCAATTTCG		
TRBV7WBL7-2;7-3	AGCTCAGGTGTGATCCAATTTCA		
TRBV7WBL7-9	CTTTCAGGTGTGATCCAATTTCT		
TRBV9	CACAACAGTTCCCTGACTTGCA		
TRBV10	CATGGGCTGAGGCTGATC		
TRBV11	CTGCAGAGAGGCTCAAAGGAGTAG		
TRBV12	AGAACCCAGGGACTCAGCTGT		
TRBV13	GAACTGAACATGAGCTCCTTGGA		
TRBV14	CTGAAAGGACTGGAGGGACGTAT		
TRBV15	CAGGAGGCCGAACACTTCTTT		
TRBV16	GCCTCCCAAATTCACCCTGTA		
TRBV18	CCAGCATCCTGAGGATCCA		
TRBV19	ACTGTGACATCGGCCCAAA		
TRBV20	AACCATGCAAGCCTGACCTT		
TRBV23	CCCTGCAGCCTGGCAAT		
TRBV24	GCTAAATTCTCCCTGTCCCTAGAGT		
TRBV25	TTCCCCTGACCCTGGAGTCT		
TRBV27	GGCTTAAGGCAGATCTACTATTCAATG		
TRBV28	GCCAGCACCAACCAGACAT		
TRBV29	AGCCGCCCAAACCTAACATT		
TRBV30	CGGCAGTTCATCCTGAGTTCT		
IFN?	CAGGTCATTCAGATGTAGCGGATAA	AGGAGACAATTTGGCTCTGCATT	TTTCTGTCACTCTCCTCTTTCCAATTCTTCAAA
T-bet	CAACACAGGAGGCGCACTGG	CCCCCTTGTTGTTTGTGAGCT	CACCTGTTGTGGTCCAAGTTTAATCAGCACC
FoxP3	TGGCAAACGGAGTCTGCAA	TCTCATCCAAGAGGTGATCTGCTT	AGCCGGGAGAGTTTCTCAAGCACTGC
GATA3	CCTCATTAAGCCCAAGCGAAG	TTGGCATTTCCTCCAGAGT	TCCTGTGCGAACTGTCAGACCACCAC
Perforin	GGACCAGTACAGCTTCAGCACTG	AGTCAGGGTGCAGCGGG	TGCCGCTTCTACAGTTTCCATGTGGTACAC
Granzyme B	GCGAATCTGACTTACGCCATTATT	CAAGAGGGCCTCCAGAGTCC	CCCACGCACAACTCAATGGTACTGTCG
B19V	CATTTTCCAGACAGTTTTTAATTCCA	CTTGCTGCGGGAGAAAACAC	ATGACCCAGAGCACC

Sequences of primers and fluorescence hybridization probes. For the different TRBV forward primers, one common reverse primer and one common hybridization probe were used. ^*^ For TRBV5, 6 and 7, wobbled (WBL) forward primers were designed.

B19V IgG and IgM titers were quantified by standard ELISA techniques according to the manufacturer's instructions (Mikrogen GmbH, Neuried, Germany). Antibodies against native and denatured PVB19 proteins were analyzed in the serum using *recom*Line® blot assays according to the manufacturer's instructions (Mikrogen GmbH) [14]. In brief, *recom*Line® strips were incubated with serum samples (20 μl each) and subsequently anti-human IgG or IgM conjugated with horseradish peroxidase (HRPO) were added. In positive HRPO reactions, a dark band developed at the corresponding locus on the strips. The positive control bands were positive in all blots performed. Reactivities of serum antibodies against the recombinant antigens were evaluated by comparison with a cut-off band. Reactivities stronger than or equivalent to the reference band were evaluated positive.

### Analysis of B19V specific T-cells

Ex-vivo analysis of the cellular immune response was performed 10 and 12 months after the initial presentation.

Anticoagulated blood (Na-citrate) was used for preparation of peripheral blood mononuclear cells (PBMCs). After separation by Biocoll-gradient centrifugation (Biochrom AG, Berlin, Germany), PBMCs were washed with sterile PBS and resuspended in RPMI 1640 medium containing 10% (v/v) fetal calf serum and 2 mM L-glutamine. The cell suspension was adjusted to a concentration of 5×10^6^ cells/ml.

T-cell responses to peptides were mapped using peptide libraries of 10-amino-acid peptides with a shift of one amino acid, in agreement with previous work [9], [10]. Peptide sequences were derived from the complete proteome of B19V strain “Berlin” [15] and complemented by the alternative 10-amino-acid peptide sequences of parvovirus described by Shade et al. [16].

The scan included 1,953 peptide sequences efficiently arranged in a cross matrix system [17] resulting in 91 peptide sub-pools with a maximum number of 40 peptides. Peptide libraries were synthesized on cellulose membranes functionalized with a glycin residue using the SPOT technology [18], resulting in 10-amino-acid peptides with an additional N-terminal glycin-amide.

For the screening with complete peptide libraries, 96 well round bottom plates containing 200 μl of the adjusted cell suspension (1×10^6^ cells) were used. Peptide sub-pools were added in 25 μl RPMI/FCS/L-Glu medium containing 1 mg/ml (final concentration) of each peptide.

Responses to sub-pools of this library were fine mapped with 10-amino-acid peptides, synthesized on solid phase support with a purity of about 99% following HPLC purification. Additionally, a 13-amino-acid peptide sequence containing two of the CD8^+^ epitope candidates with an additional CD4^+^ response was synthesized. For the fine mapping and the examination of T-cell responses at the second time point a larger number of cells were used and cell stimulating experiments were carried out in tubes. 400 μl of cell suspension (2×10^6^ cells) was transferred to Falcon 2052 tubes and peptides were added in 100 μl RPMI/FCS/L-Glu medium containing 1 μg/ml (final concentration) of each peptide. For all experiments a negative control sample was incubated with 1 μl/ml DMSO, a positive control sample with 1 μg/ml Staphylococcus Enterotoxin B (SEB). Tubes and plates were incubated in a standard incubator (5% CO_2_ humidified atmosphere). After 2 h, Brefeldin A (BFA) was added. All plate wells were supplemented with 2.5 μg of BFA in 25 μl RPMI/FCS/L-Glu and Falcon 2052 tubes with 10 μg BFA in 500 μl RPMI/FCS/L-Glu (final concentration was 10 μg/ml). After an incubation time of 16 h (i.e. 14 h after BFA addition), 10 mM EDTA was added and after an additional 10 min incubation was ended by washing with cold PBS containing 0.5% BSA and 0.1% NaN_3_. Extracellular markers were stained with fluorochrome-labeled monoclonal antibodies (mAb). Then cells were washed with PBS/FCS/NaN_3_ and lysis of erythrocytes was performed (lysing solution; Becton Dickinson). Following permeabilization (permeabilizing solution II; Becton Dickinson), intracellular staining (ICS) of cells with fluorochrome labeled mAb was performed; finally cells were washed in PBS containing 0.5% paraformaldehyde and analyzed on a LSRII flow cytometer using the DIVA software (both from Becton Dickinson).

HLA class I typing by low resolution PCR on DNA extracted from peripheral blood (tissue typing laboratory, Charité–Universitätsmedizin Berlin) revealed the presence of HLA A*02, A*11, B*07 in this patient.

SALK- and GLCP-reactive cells were enriched from PBMCs after incubation with these two peptides over 6 h using the IFNγ secretion assay according to the manufacturer's instructions (Miltenyi Biotech, Bergisch Gladbach, Germany). PBMCs were separated from peripheral blood obtained in Vacutainer® EDTA tubes (BD Biosciences, Heidelberg, Germany) using the LSM 1077 Lymphocyte Separation Medium (PAA, Pasching, Germany) density gradient. Both the positively and the negatively selected cells using the IFNγ secretion assay, as well as unselected PBMCs were lysed in RLT® buffer (Qiagen, Hilden, Germany), and total RNA was gained using the Rneasy® system (Qiagen) according to the manufacturer's instructions including DNAse treatment. Retrotranscription was carried out using the High Capacity Archive cDNA Kit® (ABI).

### Preamplified Real-time RT-PCR of SALK- and GLCP-reactive cells

We used self-designed (Table 2) and Taqman® ABI inventoried gene assays (Table 3). For the TRBV/TRBC gene assay design, we used the gene sequences published at the Immunogenetics Database [http://imgt.cines.fr/] according to ABI recommendations using the Primer Express Software (version 2.0; ABI). Sequence homologies between the various functional TRBVs were excluded by multiple alignments using ClustalX Software (version 1.83; University of Strasbourg, France). We chose TRBV specific forward primers and one common reverse TRB primer on the constant TRB (TRBC) region and a probe on the TRBC region (encompassing both human TRBC1 and TRBC2). A further gene assay was designed on the constant region of the TRBC (encompassing both human TRBC1 and TRBC2). For TRBV5, 6, and 7, wobbled (WBL) primers were designed to encompass few base differences. TRBV and TRBC minor groove binder (MGB) probes were synthesized by ABI. Regarding the remaining self-designed gene assays, we used the Oligo 4.1 software (Molecular Biology Insights, Cascade, USA) and published genomic sequences (National Center for Biotechnology Information/NCBI) according to ABI recommendations. All probes used were FAM (carboxyfluorescein) dye labeled.

**Table 3:** ABI inventoried Taqman® gene expression assays.

Gene name	ABI ID number
CD3d	Hs00174158_m1
IL2	Hs00174114_m1
IL4	Hs00174122_m1
IL6	Hs00174131_m1
IL27	Hs00377366_m1
TNFα	Hs00174128_m1
NFkB	Hs00765730_m1

ABI inventoried Taqman® gene expression assays and the respective ABI ID numbers.

cDNA was preamplified using the PreAmp® MasterMix Kit (ABI) according to the manufacturer's instructions, modified by employing half of the suggested volume of PreAmp reaction (25 μl PreAmp reaction volume). The pooled TaqMan® ABI inventoried gene assays (including fluorescent probes) as well as the forward/reverse primers of the self-designed gene assays were diluted with 1× Tris-EDTA (TE; 10 mmol/l Tris-HCl and 1 mmol EDTA, pH 7.6) buffer, so that each assay was at a final concentration of 0.2 fold in the PreAmp primer/assay pool. The PreAmp reaction involved the amplification of 1–250 ng (maximum 6.25 μl) cDNA in a 25 μl reaction consisting of 12.5 μl TaqMan® PreAmp Master Mix and 6.25 μl pooled primer/assay mix (0.2×, each assay). T-PreAmp of this primer/gene assay pool was carried for 14 cycles on a PTC-100 Programmable Thermal Controller (MJ Research, Watertown, Massachusetts, USA) with the following program: denaturation at 95°C for 10 min and 14 cycles of amplification (15 sec at 95°C, 4 min at 60°C). The preamplified products were then diluted with Tris-EDTA-buffer at a ratio 1∶20 (resulting volume 500 μl) and were used as templates for the real-time RT-PCR analysis.

The real-time RT-PCR reactions were performed in a final volume of 12.5 μl, containing 1 μl cDNA, 6.25 μl Master Mix (TaqMan™ Universal PCR Master Mix, No AmpErase® UNG, ABI), 0.21 μl probe (final concentration 0.05 μM) and 1.25 μl forward and reverse primers (final concentration 0.9 μM). Reactions were made up to a final volume of 12.5 μl with sterile water. All experiments were performed in duplicates on a 7900 HT Fast real-time PCR System (ABI). The real-time RT-PCR protocol was as following: Denaturation by a hot start at 95°C for 10 min, followed by 40 cycles of a two-step program (denaturation at 95°C for 15 sec and annealing/extension at 60°C for 1 min). TRBV gene expression was normalized to TRBC, and effector T-cell genes were normalized to CD3d applying the formula 2^−ΔCt^. mRNA expression in positively selected cells was compared with mRNA expression in negatively selected cells and unselected PBMCs.

### Ethics

These investigations were approved by the local ethics committee at the Charité–Universitätsmedizin Berlin in the framework of the Sonderforschungsbereich TR19. The investigations were in agreement with the Helsinki Declaration. Written informed consent was obtained from the patient.

### Statistical methods

Statistical analysis was performed using JMP Statistical Discovery Software V5.1.2 (SAS Institute, Inc., Cary, NC, USA). We compared ordinal with continuous data employing the student's t-test. Probability values (p) <0.05 were considered statistically significant.

## Results

**Figure 1:**
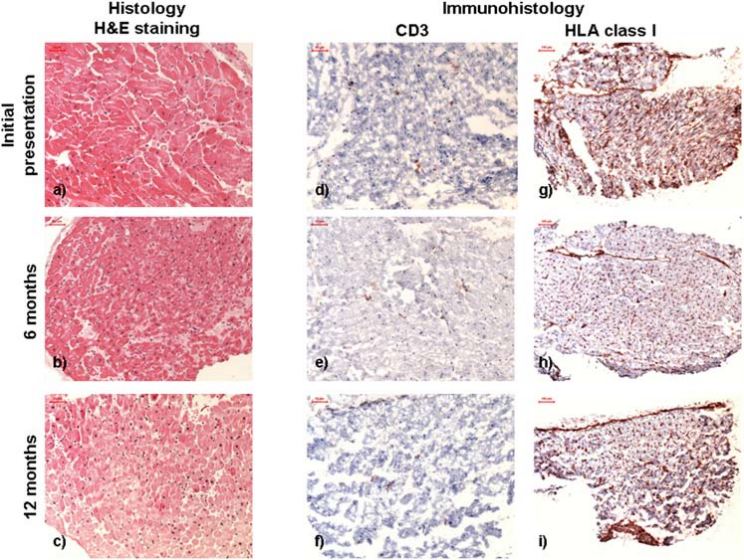
Histological (a, b, c) and immunohistological (d–i) stainings (CD3: d–f; HLA class I expression: g–i) of the EMBs at the initial presentation, at 6 and 12 months follow-up. Histological analyses did not reveal active or borderline myocarditis at any time point. The initially increased CD3^+^ T-cells and HLA class I expression decreased at the follow-up EMBs. Original magnification a–f: 200×fold. Original magnification g–i: 100×fold.

Histological analysis of EMBs revealed no evidence of active myocarditis according to the Dallas criteria [19] at any time point (Figure 1a, b, c). However, quantification of immunohistological stainings of EMBs [12] by digital image analysis (DIA) revealed increased infiltrates and expression of cell adhesion molecules (CAMs), consistent with DCMi (Table 1; Figure 1d, g). nPCR for cardiotropic viral genomes [6] confirmed positive B19V results in EMBs, PBMCs and the serum. Quantitative PCR (qPCR) revealed a B19V load of 115,091 copies/μg nucleic acids in the EMBs, and 184,000 copies/μg nucleic acids in the PBMCs, respectively. The B19V load in the serum was quantified as 57,468 viral copies/ml. B19V qPCR of EMBs 6 and 12 months after the initial presentation showed substantially decreased B19V loads (23,846 and 1,026 viral copies/μg nucleic acids, respectively). Noticeably, B19V could no longer be tracked either in PBMCs or in the serum in these follow-up analyses by nPCR. This was paralleled by a decreasing infiltration and CAMs expression in the follow-up EMBs, reaching normal values in the EMBs obtained at the third time point (Table 1; Figure 1e, f, h, i). B19V IgM were detectable at the initial presentation by both ELISA and recomLine® blots, but not at follow-up analyses, consistent with seroconversion after an acute B19 infection at the initial presentation. The B19V IgG titer remained almost stable at the three time points, however, recomLine® blot analyses revealed the occurrence of NS1-specific antibodies 6 and 12 months after the initial presentation, indicating a persisting B19V infection (Table 1).

**Figure 2:**
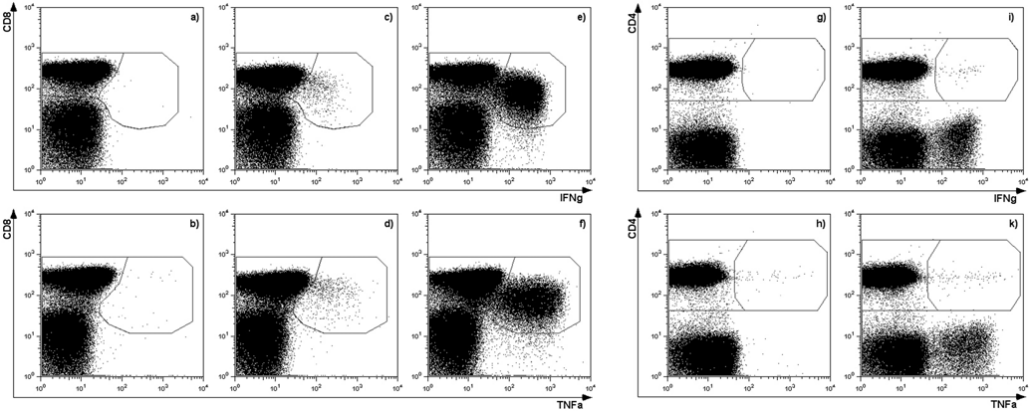
FACS analysis of two B19V NS1-specific CD8^+^ T-cell responses and one CD4^+^ T-cell response 10 months after the initial presentation. The upper panels illustrate IFNγ secretion, and the lower panels TNFα secretion, both 16 h after stimulation. Plots show negative controls (a, b, g, h), CD8^+^ T-cell responses following stimulation with the 10-amino-acid peptides GLCPHCINVG (c, d) and SALKLAIYKA (e, f), and the CD4^+^ T-cell response following stimulation with the 13-amino-acid peptide IQSALKLAIYKAT (i, k).

Ex-vivo analysis of the cellular immune response was performed 10 and 12 months after the initial presentation. Using peptide libraries representing the complete B19V proteome, strong CD8^+^ T-cell responses to at least two B19V derived peptides were identified at both time points, with larger responses measured at 10 months (Figure 2), at which time 19.7%, and 10% of the CD8^+^ T-cells responded with IFNγ secretion to the stimulation with the 10-amino-acid peptides, SALKLAIYKA_257-266_ (SALK) and QSALKLAIYK_256-265_ (QSAL), respectively. The third sequence IQSALKLAIYKAT_255-267_ (IQSA), a 13-amino-acid peptide, containing the two other sequences, activated 14.6% of the CD8^+^ T-cells. Similar frequencies were observed when using TNFα as read-out instead of IFNγ (Figure 2). The sequence shift from IQSA to QSAL and SALK is only one amino acid at a time, suggesting that they contain the same complete or partial epitope. A small but distinct CD4^+^ T-cell response (0.02–0.04% of all CD4^+^ T-cells) to each of these peptides was also detected (shown in Figure 2 for the 13-amino-acid peptide). Two additional 10-amino-acid peptides also stimulated CD8^+^ T-cell responses: The peptide GLCPHCINVG_613-622_ (GLCP) was recognized by 0.71% of CD8^+^ T-cells, and the peptide LLHTDFEQVM_276-285_ (LLHT) was recognized by 0.06% of the CD8^+^ T-cells, both inducing IFNγ. Only the GLCP peptide induced a similar TNFα response of similar size. Both sequences include a published HLA-A2 presented epitope (GLCPHCINV [20] and LLHTDFEQV [20]), in agreement with the HLA-Type of the donor. After two months, the frequencies of the above described IFNγ T-cell responses had decreased to 5.34% (SALK), 4.4% (QSAL) and 0.07% (IQSA), 0.08% (GLCP) and 0.03% (LLHT). CD4^+^ T-cells producing IFNγ in response to stimulation with GLCP, SALK and IQSA were also still detectable, however response were smaller than 0.025%.

**Figure 3:**
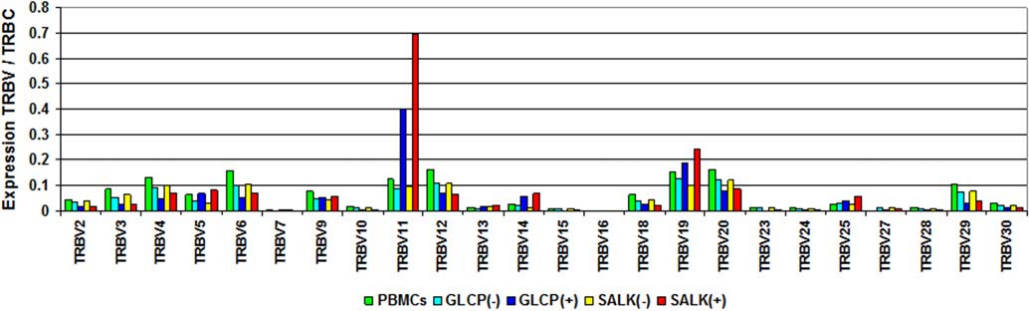
TRBV expression of B19V NS1-specific T-cells. Bars indicate the TRBV expression normalized to TRBC in non-selected PBMCs compared with positively and negatively selected GLCP and SALK reactive T-cells which were enriched using the IFNγ secretion assay.

**Figure 4:**
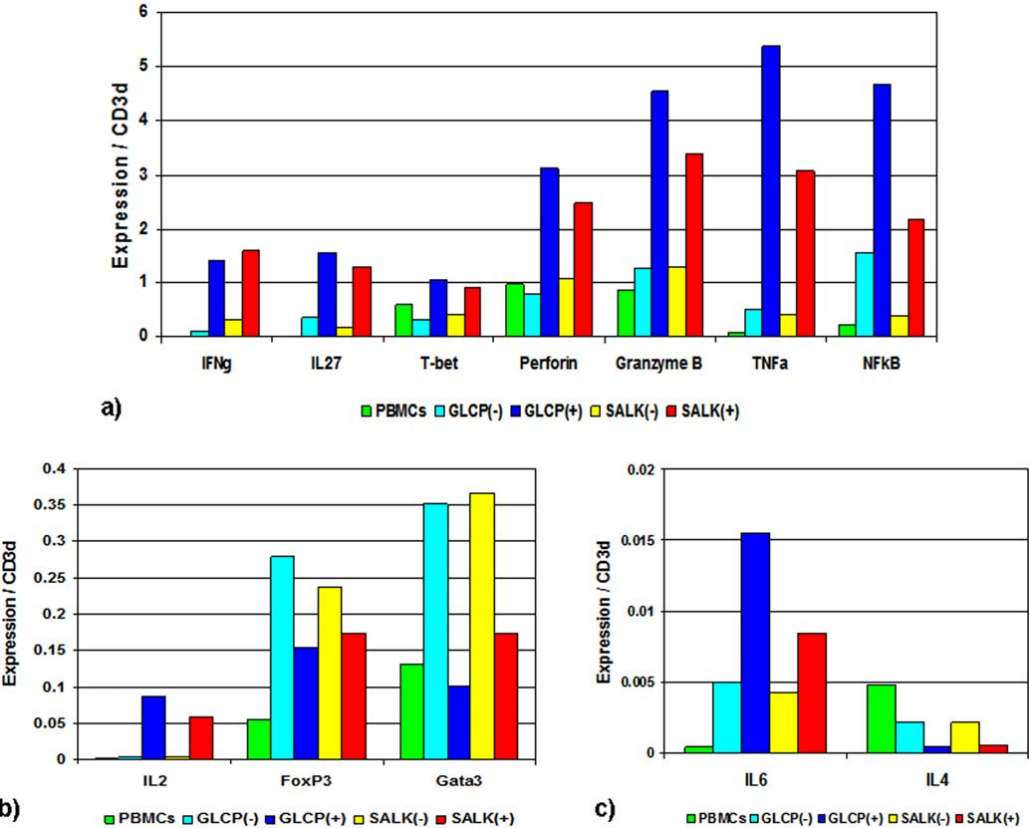
Expression of effector T-cell markers of B19V NS1-specific T-cells. The bars indicate marker expression (normalized to CD3d in non-selected PBMCs) compared with positively and negatively selected GLCP and SALK reactive T-cells enriched using the IFNγ secretion assay. The 3 panels show different target gene expression levels.

Real-time RT-PCR for functional TRBV expression in SALK- and GLCP-reactive T-cells demonstrated an over proportional TRBV11 expression compared with the respective non-reactive cells (SALK: 7.5 fold; GLCP: 4.7 fold) and non-selected PBMCs (Figure 3). Furthermore, SALK- and GLCP-reactive T-cells exhibited dominant expression of the type-1 T-cell markers, IFNγ (SALK: 11.9 fold; GLCP: 5 fold), IL2 (SALK: 16.6 fold; GLCP: 24.7 fold), IL27 (SALK: 8.7 fold; GLCP: 4.4 fold), T-bet (SALK: 2.3 fold; GLCP: 3.3 fold), and of the CTL markers, Perforin (SALK: 2.3 fold; GLCP: 4 fold) and Granzyme B (SALK: 2.6 fold; GLCP: 3.6 fold). In contrast to this, markers for type-2 (GATA3: SALK 0.4 fold and GLCP 0.3 fold, respectively; IL4: SALK 0.3 fold and GLCP 0.2 fold, respectively) and for regulatory T-cells (FoxP3; SALK: 0.7 fold and GLCP: 0.6 fold, respectively) were expressed at low levels. In addition, a substantially higher expression of general markers of inflammation was measured in the positively selected IFNγ expressing cells: IL-6 (SALK: 2.0 fold; GLCP: 3.1 fold), TNFα (SALK: 7.5 fold; GLCP: 10.7 fold) and NFkB (SALK: 5.9 fold; GLCP: 3 fold) (Figure 4).

## Discussion

This patient presented with signs and symptoms suggestive of acute myocarditis [6], [11]. DCMi was confirmed by immunohistology [12], [21], in the absence of active myocarditis according to the histological Dallas criteria [19]. This is in line with the substantially higher sensitivity of immunohistological EMBs assessment compared with the Dallas criteria [12], [22], [23]. B19V is the most commonly detected virus in patients presenting with acute myocarditis or DCM/DCMi [5], [6], [7], [8]. Usually, B19V genomes are confirmed in EMBs, but not PBMCs or sera of such patients. In this particular case, the B19V load was almost the same in EMBs and PBMCs at the initial presentation, suggesting viremia, i.e. active viral replication. While it is known that IgG-complexed virus particles can be taken up by mononuclear phagocytes via the Fc-receptor or complement-receptor mediated endocytosis, the fact that B19V genomes were detected in the serum with a quantifiable number of viral copies argued for the presence of viremia. At follow-up, however, B19V genomes were confirmed in EMBs, but not in PBMCs and serum. B19V virus load in EMBs decreased substantially over time, however, was detectable at all time points. LVEF increased and LVEDD decreased in parallel under conventional heart failure medication, while intramyocardial inflammation was found to have decreased at the second point, and was absent at the third time point. The changes of cardiac function data over time, and the EMBs findings are consistent with the hypothesis that the initial antiviral immune response resulted in a massive reduction or elimination of the virus, and subsequently contributed to an improvement of LV function and decrement of LV dilatation [24], [25]. The time course of B19V infection is also mirrored by the serological B19V findings: The B19V IgM titers at the initial presentation indicate an acute B19V infection with seroconversion at follow-up, while the occurrence of NS1-specific IgG indicate a persisting B19V infection 6 and 12 months after the acute event [14], [26].

It was previously reported that the CD8^+^ T-cell response targets multiple peptides situated mainly within the non-structural protein 1 (NS1) in adults with symptomatic B19V infection, and that these responses can persist for more than 2 years after the acute illness [20], [27]. In agreement with this, the major T-cell responses in this patient were directed at peptides in the NS1 protein. Consistent with reports on the development B19V specific cellular response in adults with an acute infection, we identified a B19V specific CD8^+^ T-cell response with multiple specificities, which gradually decreased over time [5], [10]. Since responses were measured at 10 and 12 months only, with a decrease noted at 12 months, we can only assume that peak levels were even higher than the values measured at 10 months.

These findings are consistent with previous studies, showing that the T-cell response increases despite resolution of clinical symptoms and targets mainly NS1 in patients acute B19V infections [28]). The demonstration of additional effector functions of these B19V specific T-cells was in agreement with an increased transcription of the type-1 and CTL markers in SALK- and GLCP-specific cells as shown.

These data indicate that the observed sustained CD8^+^ T-cell response is stimulated by B19V persistence or a delayed clearance of the virus in the endomyocardium. The continued presence of virus in the tissue, albeit in decreasing amounts, was a likely source for the continued antigen exposure of the CD8^+^ T-cells in this patient. High virus loads in EMBs suggests that the myocardium is a target for B19V specific T-cells.

We hypothesize that B19V antigen-specific CD8^+^ T-cells were crucial for the elimination of virus in PBMCs and the substantial decrease of B19V viral load in EMBs. The mechanism for virus elimination is not entirely clear, but most likely cytotoxicity, given the type-1 and CTL dominance found in SALK- and GLCP-specific cells.

Surprisingly, not only CD8^+^, but also CD4^+^ T-cell responses were induced by the 13-amino-acid peptide IQSALK and the sequences included in it (SALK and QSAL). These responses were initially identified with a 10-amino-acid peptide library, which is not optimum for the stimulation of CD4^+^ T-cells. Low frequencies of specific CD4^+^ T-cell responses were described previously after acute B19V infections [29]. These results, as well as ours, suggest a role in B19V elimination, and possibly for the pathogenesis of autoimmune phenomena.

An additional goal of this analysis was the characterization of B19V-reactive T-cells in regards of TRBV usage and functionality. Our data are consistent with a highly restricted TRBV repertoire of virus induced memory T-cells, and a type-1/CTL polarization of the T-cell mediated antiviral immune response [28], [30], [31]. The difference in type-1/type-2, CTL and Treg marker expression most likely results from the predominance of CD8^+^ T-cells in the sorted subset compared to the unselected or negatively selected populations, and reflects the composition of the B19V reactive population.

T-cell mediated responses are mediated by reactive TRBV families via interactions with HLA presented antigens [30]. A highly restricted TRBV repertoire of the CD8^+^ T-cell response in persistent B19V infections has been reported by Kasprowicz et al. (TRBV5.1 in HLA-A*2402-positive individuals) [31]. Our results confirm a highly restricted TRVB11 repertoire of NS1-specific CD8^+^ effector T-cells in this HLA A*02, A*11, B*07 patient with B19V associated DCMi.

B19V genomes were previously shown to persist in various human tissues, both in healthy individuals and patients [32]. As a result of this, the biological significance of the mere presence of B19V genomes in myocardial tissue without clinical evidence of progressive myocardial disease and/or evidence of an antiviral immune response is unclear, thus far. Nevertheless, the clinical importance of B19V in DCMi was illustrated in a prospective study, in which the LVEF of DCMi patients showing viral elimination at follow-up EMBs improved substantially, whereas it deteriorated over time in patients with EMBs proven viral persistence [24]. Furthermore, viral infection, including B19V, has an independent adverse prognostic impact in patients with EMBs-proven myocarditis [33]. These investigations provide indirect evidence that viral elimination is necessary for functional improvement in DCMi. If the antiviral immune response, as measured in the present study, was indeed a correlate of effective B19V elimination, such a measurement early in the course of disease may be a useful surrogate marker predicting a favorable outcome in B19V associated DCMi.

## References

[1] Cossart YE, Field AM, Cant B, Widdows D (1975). Parvovirus-like particles in human sera.. Lancet.

[2] Young NS, Brown KE (2004). Parvovirus B19.. N Engl J Med.

[3] Lehmann HW, Knoll A, Kuster RM, Modrow S (2003). Frequent infection with a viral pathogen, parvovirus B19, in rheumatic diseases of childhood.. Arthritis Rheum.

[4] Lehmann HW, von Landenberg P, Modrow S (2003). Parvovirus B19 infection and autoimmune disease.. Autoimmun Rev.

[5] Pankuweit S, Moll R, Baandrup U, Portig I, Hufnagel G (2003). Prevalence of the parvovirus B19 genome in endomyocardial biopsy specimens.. Hum Pathol.

[6] Kuhl U, Pauschinger M, Bock T, Klingel K, Schwimmbeck CP (2003). Parvovirus B19 infection mimicking acute myocardial infarction.. Circulation.

[7] Kuhl U, Pauschinger M, Noutsias M, Seeberg B, Bock T (2005). High prevalence of viral genomes and multiple viral infections in the myocardium of adults with "idiopathic" left ventricular dysfunction.. Circulation.

[8] Mahrholdt H, Wagner A, Deluigi CC, Kispert E, Hager S (2006). Presentation, patterns of myocardial damage, and clinical course of viral myocarditis.. Circulation.

[9] Kern F, Surel IP, Brock C, Freistedt B, Radtke H (1998). T-cell epitope mapping by flow cytometry.. Nat Med.

[10] Bunde T, Kirchner A, Hoffmeister B, Habedank D, Hetzer R (2005). Protection from cytomegalovirus after transplantation is correlated with immediate early 1-specific CD8 T cells.. J Exp Med.

[11] D'Ambrosio A, Patti G, Manzoli A, Sinagra G, Di Lenarda A (2001). The fate of acute myocarditis between spontaneous improvement and evolution to dilated cardiomyopathy: a review.. Heart.

[12] Noutsias M, Seeberg B, Schultheiss HP, Kuhl U (1999). Expression of cell adhesion molecules in dilated cardiomyopathy: evidence for endothelial activation in inflammatory cardiomyopathy.. Circulation.

[13] Noutsias M, Pauschinger M, Ostermann K, Escher F, Blohm JH (2002). Digital image analysis system for the quantification of infiltrates and cell adhesion molecules in inflammatory cardiomyopathy.. Med Sci Monit.

[14] Pfrepper KI, Enders M, Motz M (2005). Human parvovirus B19 serology and avidity using a combination of recombinant antigens enables a differentiated picture of the current state of infection.. J Vet Med B Infect Dis Vet Public Health.

[15] Liefeldt L, Plentz A, Klempa B, Kershaw O, Endres AS (2005). Recurrent high level parvovirus B19/genotype 2 viremia in a renal transplant recipient analyzed by real-time PCR for simultaneous detection of genotypes 1 to 3.. J Med Virol.

[16] Shade RO, Blundell MC, Cotmore SF, Tattersall P, Astell CR (1986). Nucleotide sequence and genome organization of human parvovirus B19 isolated from the serum of a child during aplastic crisis.. J Virol.

[17] Bialek K, Swistowski A, Frank R (2003). Epitope-targeted proteome analysis: towards a large-scale automated protein-protein-interaction mapping utilizing synthetic peptide arrays.. Anal Bioanal Chem.

[18] Frank R (2002). The SPOT-synthesis technique. Synthetic peptide arrays on membrane supports–principles and applications.. J Immunol Methods.

[19] Aretz HT (1987). Myocarditis: the Dallas criteria.. Hum Pathol.

[20] Norbeck O, Isa A, Pohlmann C, Broliden K, Kasprowicz V (2005). Sustained CD8+ T-cell responses induced after acute parvovirus B19 infection in humans.. J Virol.

[21] Richardson P, McKenna W, Bristow M, Maisch B, Mautner B (1996). Report of the 1995 World Health Organization/International Society and Federation of Cardiology Task Force on the Definition and Classification of cardiomyopathies.. Circulation.

[22] Noutsias M, Pauschinger M, Poller WC, Schultheiss HP, Kuhl U (2003). Current insights into the pathogenesis, diagnosis and therapy of inflammatory cardiomyopathy.. Heart Fail Monit.

[23] Baughman KL (2006). Diagnosis of myocarditis: death of Dallas criteria.. Circulation.

[24] Kuhl U, Pauschinger M, Seeberg B, Lassner D, Noutsias M (2005). Viral persistence in the myocardium is associated with progressive cardiac dysfunction.. Circulation.

[25] Liu PP, Mason JW (2001). Advances in the understanding of myocarditis.. Circulation.

[26] Modrow S, Dorsch S (2002). Antibody responses in parvovirus B19 infected patients.. Pathol Biol (Paris).

[27] Mitchell LA, Leong R, Rosenke KA (2001). Lymphocyte recognition of human parvovirus B19 non-structural (NS1) protein: associations with occurrence of acute and chronic arthropathy?. J Med Microbiol.

[28] Isa A, Kasprowicz V, Norbeck O, Loughry A, Jeffery K (2005). Prolonged activation of virus-specific CD8+T cells after acute B19 infection.. PLoS Med.

[29] Kasprowicz V, Isa A, Tolfvenstam T, Jeffery K, Bowness P (2006). Tracking of peptide-specific CD4+ T-cell responses after an acute resolving viral infection: a study of parvovirus B19.. J Virol.

[30] Kaech SM, Hemby S, Kersh E, Ahmed R (2002). Molecular and functional profiling of memory CD8 T cell differentiation.. Cell.

[31] Kasprowicz V, Isa A, Jeffery K, Broliden K, Tolfvenstam T (2006). A highly restricted T-cell receptor dominates the CD8+ T-cell response to parvovirus B19 infection in HLA-A*2402-positive individuals.. J Virol.

[32] Norja P, Hokynar K, Aaltonen LM, Chen R, Ranki A (2006). Bioportfolio: lifelong persistence of variant and prototypic erythrovirus DNA genomes in human tissue.. Proc Natl Acad Sci U S A.

[33] Caforio AL, Calabrese F, Angelini A, Tona F, Vinci A (2007). A prospective study of biopsy-proven myocarditis: prognostic relevance of clinical and aetiopathogenetic features at diagnosis.. Eur Heart J.

